# Low-temperature synthesis of CuO-interlaced nanodiscs for lithium ion battery electrodes

**DOI:** 10.1186/1556-276X-6-397

**Published:** 2011-05-26

**Authors:** Seung-Deok Seo, Yun-Ho Jin, Seung-Hun Lee, Hyun-Woo Shim, Dong-Wan Kim

**Affiliations:** 1 Department of Materials Science and Engineering, Ajou University, Suwon 443-749, Korea

## Abstract

In this study, we report the high-yield synthesis of 2-dimensional cupric oxide (CuO) nanodiscs through dehydrogenation of 1-dimensional Cu(OH)_2 _nanowires at 60°C. Most of the nanodiscs had a diameter of approximately 500 nm and a thickness of approximately 50 nm. After further prolonged reaction times, secondary irregular nanodiscs gradually grew vertically into regular nanodiscs. These CuO nanostructures were characterized using X-ray diffraction, transmission electron microscopy, and Brunauer-Emmett-Teller measurements. The possible growth mechanism of the interlaced disc CuO nanostructures is systematically discussed. The electrochemical performances of the CuO nanodisc electrodes were evaluated in detail using cyclic voltammetry and galvanostatic cycling. Furthermore, we demonstrate that the incorporation of multiwalled carbon nanotubes enables the enhanced reversible capacities and capacity retention of CuO nanodisc electrodes on cycling by offering more efficient electron transport paths.

## Introduction

Inexpensive, environmentally innocuous, and easily producible cupric oxide (CuO) is an important p-type semiconductor with a bandgap of 1.2 eV that is widely studied in applications, including catalysts, gas sensors, photoconductive/photochemical cells, and other electronic devices [1-5]. Additionally, a great effort has recently been applied to the nanostructuring of CuO as it can deliver much higher reversible capacities than commercial graphite-based electrodes through the conversion reaction with Li (CuO + 2e^- ^+ 2Li^+ ^↔ Cu^0 ^+ Li_2_O). Thus, various CuO nanostructures (nanoparticles, nanowires, nanorods, nanotubes) have been shown to be good candidates as electrodes for lithium ion batteries [6-8]. Zhang *et al. *reported the size dependency of the electrochemical properties in zero-dimensional CuO nanoparticles synthesized by thermal decomposition of CuC_2_O_4 _precursor at 400°C [9]. One-dimensional (1-D) CuO nanorod and nanowire CuO electrodes have also been produced *via *hydrothermal and wet chemical methods for enhanced reversible capacity [10,11]. Recently, two-dimensional (2-D) CuO nanoribbons and other three-dimensional hierarchical nanostructures such as dendrites and spheres, assembled with nanoneedles, have been reported as high-performance anodes for Li ion batteries [12-14].

Herein, we demonstrate a low-temperature and large-scale conversion of initially prepared 1-D Cu(OH)_2 _nanowires into 2-D CuO nanodiscs and further vertically interlaced nanodisc structures. The detailed morphological evolution during the growth of the nanostructured CuO was examined by controlling the reaction conditions, such as synthesis time and temperature. The electrochemical reaction of Li with the obtained CuO nanodiscs was investigated by cyclic voltammetry (CV) and galvanostatic cycling. Furthermore, the enhanced reversible capacities and capacity retention in the CuO nanodisc composite electrodes, by the incorporation of multiwalled carbon nanotubes (MWCNTs), are reported by offering better efficient electron transport paths.

## Experimental

Cu(OH)_2 _nanowire precursors were prepared by a simple chemical solution route at room temperature [15]. First, 30 mL of 0.15 M NH_4_OH (28-30% as ammonia, NH_3_, Dae-Jung Chemical, Shiheung, South Korea) was added to 100 mL of 0.04 M copper (II) sulfate pentahydrate (CuSO_4_·5H_2_O, 99.5%, JUNSEI Chemical, Tokyo, Japan), followed by drop-wise addition of 6.0 mL of 1.2 M NaOH (98%, Dae-Jung Chemical, Shiheung, South Korea) under magnetic stirring. The Cu(OH)_2 _precipitate appeared in the blue solution. The as-prepared solution containing the Cu(OH)_2 _precursor was stored at room temperature for 1 h and heat-treated at 60°C for 3 h in a convection oven to produce CuO nanostructures. The black powders were centrifuged and washed with deionized water and ethanol several times and were dried overnight at 70°C in a vacuum oven.

For preparation of the multiwalled carbon nanotube (MWCNT)/CuO composites, a calculated amount (60 mg) of synthetic multiwalled carbon nanotubes (CNT Co., Ltd., Incheon, South Korea) was first dispersed and sonicated for 3 h in 100 mL deionized water in the presence of cetyltrimethylammonium bromide (CTAB, 99%, 0.2 mg, Sigma-Aldrich, Saint Louis, MO, USA) [16]. After complete dispersion of the MWCNTs, the same steps as those for the CuO nanopowders were followed.

The crystal structures and morphologies of each powder were investigated using X-ray powder diffraction (XRD; model D/MAX-2500V/PC, Rigaku, Tokyo, Japan), field emission scanning electron microscopy (FESEM; model JSM-6330F, JEOL, Tokyo, Japan), and high-resolution transmission electron microscopy (HRTEM; model JEM-3000F, JEOL, Tokyo, Japan). Additionally, the specific surface areas were examined using the Brunauer-Emmett-Teller (BET; Belsorp-mini, BEL Japan Inc., Osaka, Japan) method with a nitrogen adsorption/desorption process.

The electrochemical performance of each powder was evaluated by assembling Swagelok-type half cells, using a Li metal foil as the negative electrode. Positive electrodes were cast on Cu foil by mixing prepared powders (1.0-2.0 mg) with Super P carbon black (MMM Carbon, Brussels, Belgium) and the Kynar 2801 binder (PVdF-HFP) at a mass ratio of 70:15:15 in 1-methyl-2-pyrrolidinone (NMP; Sigma-Aldrich, St. Louis. MO, USA). A separator film of Celgard 2400 and liquid electrolyte (ethylene carbonate and dimethyl carbonate (1:1 by volume) with 1.0 M LiPF_6_, Techno Semichem Co., Ltd., Seongnam, South Korea) was also used. The assembled cells were galvanostatically cycled between 3.0 and 0.01 V using an automatic battery cycler (WBCS 3000, WonaTech, Seoul, South Korea). All cyclic voltammetry measurements were carried out at a scanning rate of 0.1 mV s^-1^.

## Results and discussions

**Figure 1:**
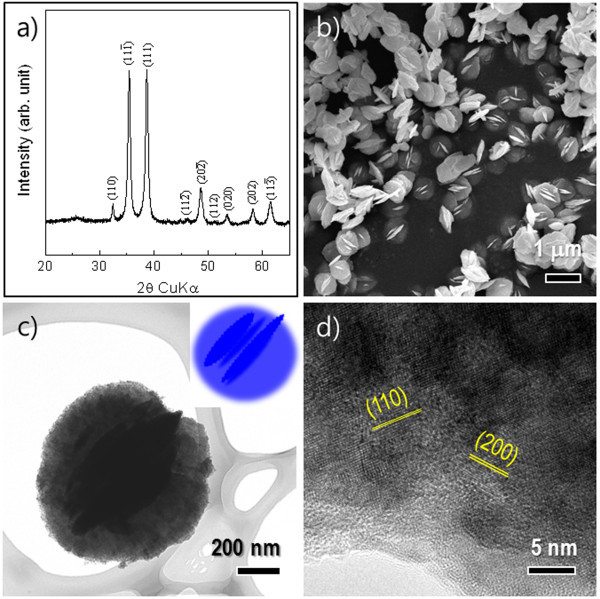
**Crystal structures of CuO products**. **(a-b) **XRD pattern and FESEM image of the CuO powders, respectively. **(c-d) **Low magnification TEM and HRTEM images of an individual interlaced nanodisc, respectively. Inset in (c) shows a schematic illustration emphasizing the interlaced disc structure.

The crystal structures of the obtained CuO products were analyzed through the XRD patterns in Figure 1a. All the reflection peaks could be completely indexed as well-crystalline, monoclinic CuO, which was in good agreement with literature values (JCPDS file no. 48-1548). As shown in Figure 1a, no characteristic peaks from unreacted starting materials or initially synthesized Cu(OH)_2 _precursors were detected on the XRD patterns of the products, indicating that all samples obtained were single-phase CuO.

Figure 1b shows the low magnification FESEM image of CuO powders. It can be clearly observed that uniform 2-D disc-like morphologies with an average diameter of 500-700 nm and a thickness of 30-50 nm were obtained on a large scale. More interestingly, more than one standing disc was inserted into the central part of the lying discs, indicating CuO-interlaced nanodisc structures. This characteristic nanostructure was also confirmed by local contrast differences in a representative transmission electron microscopy (TEM) image of an individual disc (Figure 1c). The inset in Figure 1c depicts a typical CuO-interlaced nanodisc based on the FESEM and TEM observations. Figure 1d shows the magnified HRTEM image of the surface region in the nanodisc. The measured lattice spacings obtained from the HRTEM image were 2.76 and 2.30 Å, in accordance with the (110) and (200) planes of the monoclinic CuO structure, respectively.

**Figure 2:**
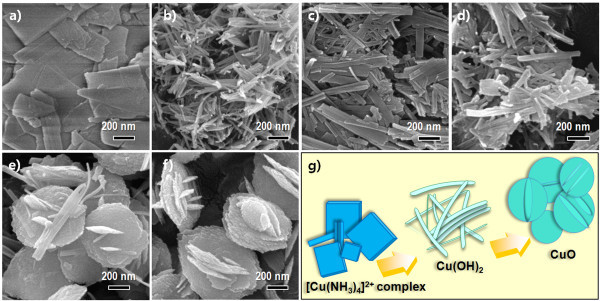
**FESEM images**. **(a) **[Cu(NH_3_)_4_]^2+ ^complex, **(b) **Cu(OH)_2 _nanowires at room temperature, **(c-d) **Cu(OH)_2 _nanowires after reaching 40°C and 50°C, respectively. **(e-f) **CuO-interlaced nanodiscs at 60°C after 0 and 3 h, respectively. **(g) **Schematic diagram of the morphology evolution steps for CuO nanostructures.

To understand the growth mechanism of the above CuO-interlaced nanodisc structures, temperature- and time-dependent experiments were carried out. Figure 2 shows the series of typical FESEM images of samples taken after reaching a preset temperature and time. First, Cu^2+ ^ions in the CuSO_4 _solution formed a square-planar complex [Cu(NH_3_)_4_]^2+ ^upon addition of NH_3_OH at room temperature [17]. When NaOH was further added, Cu(OH)_2 _nanocrystals began to precipitate. The template-free formation of a 1-D nanowire morphology with a 30- to 50-nm diameter was due to the specific crystal structure of Cu(OH)_2 _(Figure 2b), because the growth of the layer-structured orthorhombic Cu(OH)_2 _along [100] was much faster than along any other direction, leading to a tendency to form a 1-D structure [10,14,15,18]. With the increase in the reaction temperature from room temperature to 50°C, each nanowire was shortened and thickened laterally due to the oriented attachment of the Cu(OH)_2 _nanowires (Figure 2c,d) [17-20]. Meanwhile, a gradual dehydration involving conversion from Cu(OH)_2 _to CuO might occur.

After achieving a temperature of 60°C, most morphology changed suddenly to a disc shape by the acceleration of the oriented attachment (Figure 2e) because this 2-D compact nanostructure would be energetically favorable by reducing the interfacial energy of the 1-D nanowires [18,21]. In addition, Cu(OH)_2 _almost completely transformed into CuO. However, a small amount of the Cu(OH)_2 _phase remained, supported by the presence of nanowires reminiscent of the Cu(OH)_2 _precurso.r With a reaction time extended to 3 h, complete conversion to CuO was observed using XRD (Figure 1a).

Another feature in this CuO nanostructure was the interlaced nanodisc morphologies, namely the vertically interconnected structure with standing nanodiscs in the center part of the lying nanodiscs (Figure 2f). The morphological evolution of each intermediate phase is schematically illustrated in Figure 2g. As a detailed transformation process from Cu(OH)_2 _to CuO suggested by Cudennec *et al. *[22], the possible formation mechanism of the interlaced disc nanostructures can be suggested *via *a different dissolution and recrystallization pathway, which can be supported by the coexistence of CuO nanodiscs and Cu(OH)_2 _nanowires (Figure 2e) [23]. As the reaction time was prolonged, a Cu(OH)_2 _with a different dissolution rate, resulting in a different nucleation rate and secondary nucleation, may occur at high-energy sites on the surface of the primary nanodiscs [4]. Finally, one or more secondary standing nanodiscs gradually evolved into the larger lying flat nanodiscs, finally forming interlaced disc nanostructures, as reported in similar CuO nanostructures, by hydrothermal conversion from Cu(OH)_2 _at 100-130°C [23,24]. Therefore, the formation mechanism of the CuO-interlaced nanostructures during the phase conversion from Cu(OH)_2 _can be given *via *combined effects of the oriented attachment and subsequent dissolution-precipitation processes.

**Figure 3:**
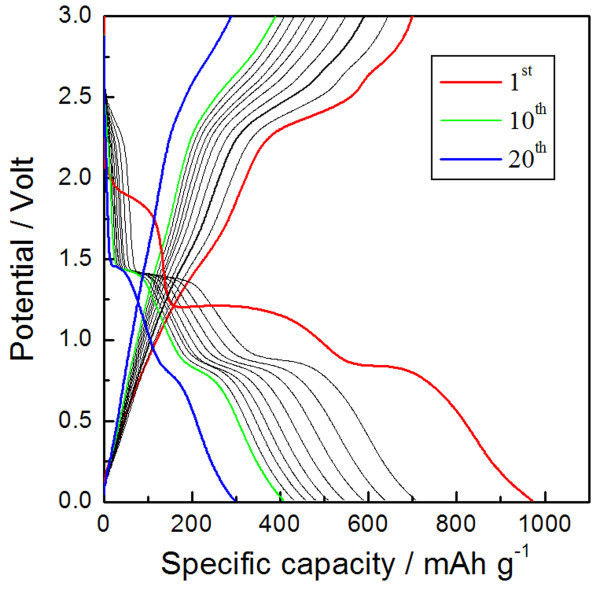
**Voltage profiles of CuO**. Galvanostatic discharge/charge voltage profiles of CuO-interlaced nanodiscs at a rate of C/5.

The galvanostatic cycling characteristics of CuO-interlaced nanodiscs in the configuration of the CuO/Li half cell were investigated over a 0.01- to 3.0-V window at a rate of C/5 (based upon a theoretical capacity of 670 mA h g^-1 ^by the conversion reaction, CuO + 2e^- ^+ 2Li^+ ^↔ Cu^0 ^+ Li_2_O), as shown in Figure 3. The first discharge and charge capacities were 971 and 699 mA h g^-1^, respectively. However, the capacity faded gradually from the subsequent cycle to a reversible capacity of 290 mA h g^-1 ^after 20 cycles. Recently, Xiang *et al. *reported the synthesis of shuttle-shaped CuO particles with a length of 1 μm and a thickness of 100-200 nm at 90°C using Cu(Ac)_2_·H_2_O precursor, which have similar structures to our CuO-interlaced nanodiscs [8]. We found that shuttle-shaped CuO (cycled at a rate of C/10) and our CuO-interlaced nanodiscs (cycled at a rate of C/5) showed similar electrochemical performance. The BET surface area of CuO-interlaced nanodiscs was estimated to be a relatively large value, approximately 60 m^2 ^g^-1^, but a significant impact on the electrochemical performance of this CuO-nanostructured electrode cannot be fully realized, possibly due to the aggregated CuO nanostructure (Figure 2f) and inhomogeneous mixing of conducting Super P carbon black with CuO nanostructures, which eventually increased the interparticle resistance, thereby degrading electrochemical performance [16,25,26]. This detrimental phenomenon may also have been caused by the significant volume change upon cycling [27].

**Figure 4:**
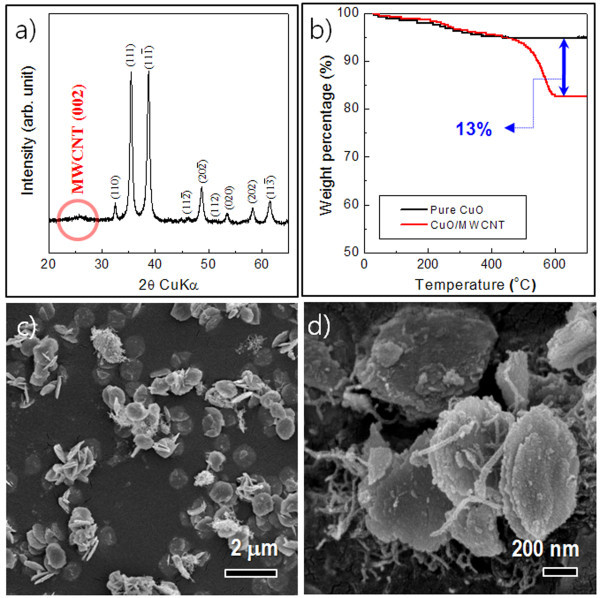
**XRD pattern of the CuO/MWCNT composites**. **(a) **XRD pattern of the CuO/MWCNT composite nanostructures. **(b) **TGA of pure CuO and CuO/MWCNT composite nanostructures. **(c-d) **Typical FESEM images of the CuO/MWCNT composite nanostructures.

Formation of composites by incorporation of MWCNTs can provide an enhanced electronic conductivity of electrodes and elastic buffers for releasing the strain of CuO during the Li conversion reaction [28]. Figure 4a shows the XRD pattern of the CuO/MWCNT composites. Compared to the XRD pattern of pure CuO-interlaced nanodiscs (Figure 1a), that of the CuO/MWCNT composites showed an additional peak at 25° by the MWCNT phase. From a comparison of the weight loss between pure CuO and CuO/MWCNT composites using a thermogravimetric analyzer (TGA), the incorporated amount of MWCNT in the composites corresponded to approximately 13%, as shown in Figure 4b.

Figure 4c,d shows typical FESEM images of the CuO/MWCNT composite. MWCNTs were spatially dispersed in the composites without any appreciable agglomeration. In addition, the morphology of CuO in the composites was found to be mostly primary nanodiscs, not the interlaced disc nanostructures. It is believed that incorporation of MWCNT mitigated secondary nucleation and growth on the surface of the primary nanodiscs.

**Figure 5:**
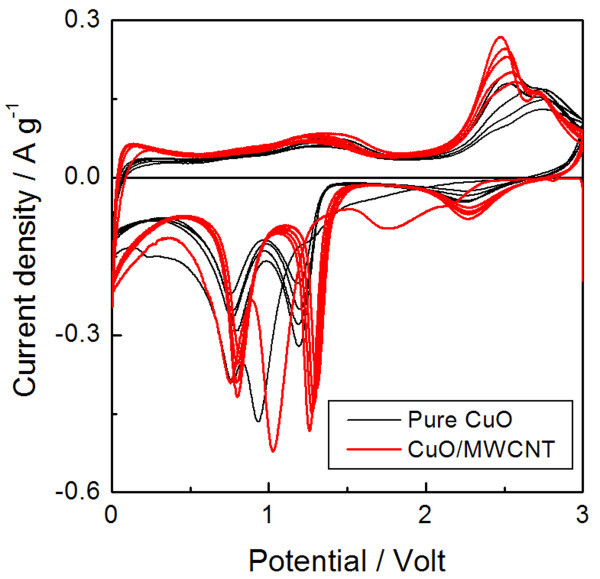
**Cyclic voltammetry for pure CuO and CuO/MWCNT**. Cyclic voltammetry of pure CuO and CuO/MWCNT composite nanostructures in the first ten cycles.

Cyclic voltammetry was recorded for pure CuO and CuO/MWCNT, as shown in Figure 5. For both samples, the CV profiles were nearly identical to those reported for the CuO nanostructures [10,12]. Efficient electron transport by introducing MWCNT upon lithiation of the CuO was confirmed by the enhanced redox peaks in the CV curves (measured on samples of similar mass at the same voltage sweep rate). Therefore, it is believed that MWCNT improved the Li electroactivity of the CuO nanostructures because of its effect on conductivity and the efficient electron path [16,26].

**Figure 6:**
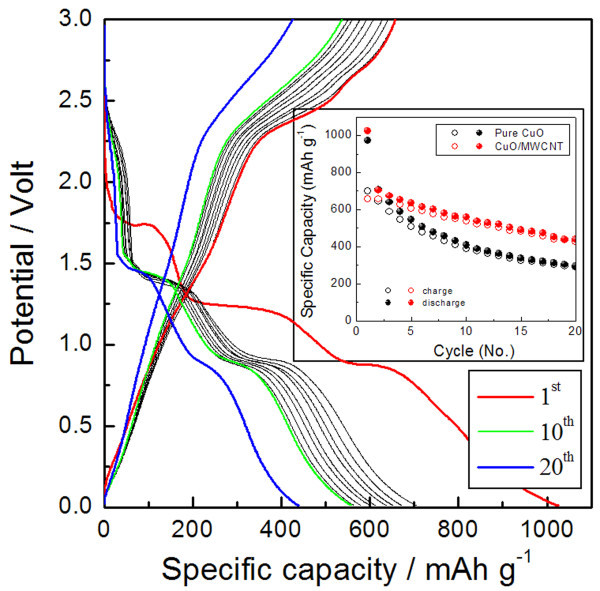
**Charge-discharge behavior of CuO/MWCNT composite electrodes**. Galvanostatic discharge/charge voltage profiles of CuO/MWCNT composite nanostructures at a rate of C/5. Inset shows the comparison of specific capacities in pure CuO and CuO/MWCNT composite nanostructures.

Figure 6 represents the charge-discharge behavior of CuO/MWCNT composite electrodes at a rate of C/5. The first discharge and charge capacities were 1,025 and 657 mA h g^-1^, respectively, and a high reversible capacity of approximately 440 mA h g^-1 ^obtained after 20 cycles. These CuO/MWCNT composite nanostructures exhibited a higher reversible lithium storage capacity and better capacity retention than the pure CuO nanodiscs (Figure 3). The specific capacity of the CuO/MWCNT composites was estimated to be 47% greater than that of pure CuO nanodiscs. This additional lithium storage capacity in the CuO/MWCNT composites may result from the efficient electron transport by the incorporation of MWCNT in high surface area CuO nanostructures. Therefore, other surface modifications using carbon or conductive metals could possibly further improve electrochemical performance of these CuO nanostructures.

## Conclusion

In summary, the successful low-temperature synthesis of phase-pure 2-D CuO-interlaced nanodiscs was demonstrated using simple dehydrogenation of 1-D Cu(OH)_2 _nanowires at 60°C in solution. The details of the growth aspects of the CuO-interlaced nanodiscs were suggested by the combined effects of the oriented attachment and subsequent dissolution-precipitation processes based on systematic temperature- and time-dependent morphology evolutions. These CuO nanostructures had a large surface area, approximately 60 m^2 ^g^-1^, and the effects of their enhanced active sites by nanostructuring on the electrochemical performance of CuO could be further realized by the incorporation of MWCNTs.

## Competing interests

The authors declare that they have no competing interests.

## Authors' contributions

S-DS carried out the CuO and CuO/MWCNT sample preparation and drafted the manuscript. Y-HJ, S-HL, and H-WS participated in microstructural and electrochemical analyses. D-WK designed the study, lead the discussion of the results and participated in writing the manuscript. All authors read and approved the final manuscript.
